# Are Anxiety and Depression Taking Sides with Knee-Pain in Osteoarthritis?

**DOI:** 10.3390/jcm11041094

**Published:** 2022-02-18

**Authors:** Matthias Vogel, Marius Binneböse, Christoph H. Lohmann, Florian Junne, Alexander Berth, Christian Riediger

**Affiliations:** 1 Department of Psychosmatic Medicine and Psychotherapy, Otto-von-Guericke-University of Magdeburg, 39120 Magdeburg, Germany; marius.binneboese@med.ovgu.de (M.B.); florian.junne@med.ovgu.de (F.J.); 2 Department of Orthopedic Surgery, Otto-von-Guericke-University of Magdeburg, 39120 Magdeburg, Germany; christoph.lohmann@med.ovgu.de (C.H.L.); alexander.berth@med.ovgu.de (A.B.); christian.riediger@med.ovgu.de (C.R.)

**Keywords:** osteoarthritis, knee-pain, lateralization, somatization, anxiety

## Abstract

Introduction: Total knee arthroplasty (TKA) bears a potential of rendering patients unsatisfied with the operation as a result of negative affectivity related to osteoarthritis and TKA. Not only is pain a lateralized experience, but negative affect and other psychosomatic correlates of pain might also be processed on grounds of lateralization. Lateralization in this context is likely linked to the amygdalae, which display differential left/right patterns of association with psychopathology. What is noteworthy is that osteoarthritis itself is linked not only to negative effects but also to childhood abuse. Method: The present study tests lateralization of psychosomatic correlates of knee-pain using the brief symptom inventory-18 (BSI-18), the dissociative experiences scale (FDS-20), the pain catastrophizing scale (PCS), the Tampa scale of kinesiophobia (TSK), the childhood trauma screener (CTS) and WOMAC. More precisely, we were interested in predicting the side of operations by means of the above-mentioned constructs using binary logistic regression, based on 150 participants (78 left knees) awaiting TKA for knee-osteoarthritis. Results: Somatization (*p* = 0.003), childhood abuse (*p* = 0.04) and pain-catastrophizing (*p* = 0.04) predicted operations on the right side. Anxiety (*p* = 0.001) and kinesiophobia (*p* = 0.002) predicted operations on the left side. Conclusions: Knee-pain may be differentially modulated by its psychosomatic correlates as a result of lateralization and corresponding patterns of psychosomatic reagibility.

## 1. Introduction

One inherent characteristic of peripheral pain is its lateralization to the hemisphere contralateral to the side of pain sensation. Lateralization is evolutionarily connected to the increase in brain size and considered a means of efficiency in processing lateralized functions as it saves extra conduction time necessary to integrate information bihemispherically [1]. Moreover, Karolis et al. [1] reported functional hemispheric asymmetry to involve less connection with the opposite hemisphere. Thus, these authors accumulate proof of the theory of interhemispheric independence [2], which is based on the assumption of reduced callosal connectivity. The corpus callosum is the main interhemispheric connection [1] and, therefore, should be considered the most important gate to the opposite hemisphere. Similarly to other brain structures, c. callosum displays plasticity, for example, in connection with biographic features such as Mehta et al. [3] found regarding the amygdalae and the hippocampus in addition to the corpus callosum. Studying individuals with a saddening record of deprivation, they found this sort of childhood adversity associated with decreased size of the left amygdala and reached the conclusion that the amygdalae are sensitive for early deprivation. The amygdalae represent an important relay for sensory inputs on their way to the contralateral prefrontal cortex [4]. Early findings on lateralization have inspired the valence hypothesis, which posits that the left hemisphere mainly processes positive emotions, and the right hemisphere predominantly negative emotions [5]. Thus, the processing of emotions is regarded as fundamentally asymmetrical [6], but this pattern may change under the influence of glucosteroids. In addition to stress, another factor contributing to the plasticity of lateralization is gender, which is associated with different functions of the amygdalae [7,8] due to the influence of sex hormones. However, another hypothesis surrounding lateralization is the right hemisphere hypothesis, which posits a preponderance for all emotions of the right hemisphere [9]. All these theories would suggest that the projection of pain to the contralateral hemisphere is linked to differential pathways of neurotransmission, including distinct patterns of affectivity-related cotransmission. The latter aspect may be of salient importance for lateralized pain, because chronic pain is particularly associated with a variety of psychosomatic constructs such as depression, anxiety, specific fear (e.g., fear of movement), somatization or posttraumatic psychopathology such as dissociation (i.e., the disintegration of certain functions including identity, perception and consciousness) [10].

All these phenomena are possibly also lateralized, similarly to what numerous findings suggest. For example, Atmaca et al. [11] reported greater right than left volumes of the amygdala in somatization, and Hardee et al. [12] reported greater left amygdala activity in response to fearful stimuli. Likewise, the right hemisphere was found to be involved in depression [13] and PTSD [14] rather than the left one.

However, the assumption of leftward lateralization of anxiety is not congruent with the predominance of the right amygdala in emotion processing [15]. In addition, Min and Lee [16] reported that the right hemisphere is more often associated with emotional reactions of somatoform symptoms. Another fundamental hint on systematic patterns of hemispheric association of pain and psychopathology stems from the observation of a heightened prevalence of pain disorders in anxiety and depression as well as in hysterical disorders that are historically reflective of somatization or conversion and dissociation, respectively [17]. Moreover, there has been reports on a general dominance of the left side as the side of pain in patients with a psychiatric diagnosis [17]. More precisely, this population is bound to prescriptions of antidepressants that mitigate pain as well. However, as Roelofs et al. [18] have noted, the lateralization of conversion disorder is controversial and dissociation may, therefore, be processed bihemispherically.

The mechanisms resulting in the lateralization of emotional transmissions are not fully understood; however, they are believed to include differences in nociceptive input, neuronal properties and control by other areas [15]. Banks et al. [19] also suggested top-down inhibition from prefrontal cortical areas, an inverse coupling of the prefrontal cortex with the amygdala, such as those observed in imaging studies in association with aversive and other emotional stimuli [19]. Moreover, the left amygdala is reported to be involved in reappraisal processes [19], as opposed to the maintenance of the affective state that is linked to the right amygdala instead. As to posttraumatic symptoms, they have been linked to right brain structures [20]. Accordingly, there are reports of an association between trauma, more precisely childhood neglect, with the volume of the right amygdala [21]. The same study reports an association between deprivation of care and anxiety, mediated by right amygdala volumes in boys. Similarly to childhood trauma, its associations with a variety of psychiatric or psychosomatic disorders could be traced back to structural changes in stress-related brain structures, which may then become maladaptive in adult environments and make individuals more vulnerable to psychiatric disorders [22,23]. For example, childhood emotional abuse was reported to be associated with reduced right amygdala volume, and Mutluer reported a reduced size of the right amygdala in PTSD based on childhood abuse [20].

The psychosomatics of knee-pain are well studied (e.g., [24]), and osteoarthritis (OA) itself is associated with depression and anxiety [25], as well as with childhood trauma [26]. Moreover, it is associated with fear avoidance causing pain to be maintained and intensified by specific fears, such as pain-catastrophizing and kinesiophobia [27]. These fears are possibly related to dissociation, which represents post-traumatic symptomatology and maybe a facet of negative affect [27], despite apparently being lateralized to the left hemisphere [28]. Generally speaking, fears are possibly subject to right hemispheric lateralization [29], at least inasmuch as trait anxiety is involved and unless in the presence of arousal. The latter is apparently associated with right hemispheric processing of emotions, such as Bourne and Vladeanu’s [29] report. Allen et al. [4] ascribed the greatest capacity of CNS for left and right differences in input and output to the amygdalae. With respect to knee osteoarthritis, neuroimaging studies have revealed greater activation of the right amygdala in response to experimentally evoked pain [30]. Accordingly, Allen et al. [4] have underscored the potential importance of hemispheric lateralization of the amygdala with respect to pain, calling for progress in this new field of study (p. 11). This led us to reanalyze a previously analyzed dataset focusing on lateralization, hypothesizing the latter as regards negative affect in patients with knee-osteoarthritis prior to total knee arthroplasty (TKA).

## 2. Materials and Methods

The analysis is based on 150 patients with primary TKA for OA of the knee, 95 (59.4%) were females. The mean age was 64.81 (10.61). The left knee was the affected side in 78 cases, whereas 72 cases involved the right knee. As we were interested in the matters of lateralization in connection with osteoarthritis-related knee pain rather than postoperative pain, we only included preoperative pain-levels in the present study.

Data were collected only 1 to 2 day(s) before the operation based on written informed consent from all participants. The study was approved by the local Institutional Review Board (177/16).

Knee pain and function were assessed using the Western Ontario and McGill Universities arthrosis index pain and function subscales (WOMAC A and WOMAC C), i.e., the Likert version in the format of a numerical rating scale ranging from 0 to 10. Cronbach’s α of the WOMAC range from 0.8 to 0.96 and its psychometric properties were judged to be good [31].

The brief symptom inventory-18 (BSI-18), a short version of the symptom check list 90, assesses symptoms of depression, anxiety and somatization. Internal consistency for the subscales ranges between 0.79 and 0.91, discriminant and convergent validity are deemed good [32]. Moreover, BSI-18 is deemed useful as a screening for psychological distress in physically ill populations [33].

The Fragebogen zu dissoziativen Symptomen-20 (FDS) [34] represents the German version of the dissociative experiences scale, of which we used the short form (FDS-20). FDS-20 is composed of the most sensitive items of the longer version on the condition that they reach a Cronbach’s α of at least 0.9. The total scale has good internal consistency (α = 0.93). Items are rated in terms of frequency on a scale ranging from never present (0%) to always present (100%). In addition, FDS-20 is suitable for the use clinical studies involving participants with chronic pain [27].

The pain catastrophizing scale (PCS) is a 13-item rating-scale comprising the subscales rumination, magnification and helplessness [35]. PCS is assigned good psychometric properties [36] and widely used in clinical populations with chronic pain.

The Tampa scale of kinesiophobia (TSK) is a thirteen-item scale rated on a 4-point Likert scale. Assessing the fear of movement and reinjury, it is a valid and reliable instrument with Cronbach’s α being 0.73 for its German version [37].

### Statistical Methods

We used *t*-testing for the descriptive comparison of groups with left/right sided knee-pain based on the variables of interest, and we used binary logistic regression analysis to predict the side of the operation, choosing the above-mentioned (preoperative) WOMAC (pain and function) and psychometric scales and subscales as predictors controlling for gender as a covariate. Statistics were computed by means of SPSS 26.

## 3. Results

Table 1 shows the means (SD) of the applied measures for the groups with left-sided and right-sided knee pain, along with *t*-testing statistics. No significant differences were observed between these groups, except with regards to childhood emotional neglect, which was more pronounced in candidates for left-sided surgery. Table 2 displays the results of binary logistic regression analyses. Coefficient B reveals the status of laterality according to the values assigned to the left (0) and the right (1) knee, respectively. A nagative exponent B, therefore, refers to the left side, and vice versa. The binomial logistic regression model was statistically significant (χ^2^ = 27.98; *p* = 0.001), resulting in a moderate amount of explained variance [38], as shown by Nagelkerke’s R^2^ = 0.31. The levels of pain and psychopathology did not differ significantly between left-sided and right-sided knee-OA. Most importantly, however, this study shows predictions of right-sided pain by somatization, childhood physical abuse and pain catastrophizing and of left-sided pain by anxiety and kinesiophobia.

## 4. Discussion

We sought to analyze laterality in psychosomatic correlates of osteoarthritis-linked knee pain and report right-sided pain to be associated with somatization, childhood physical abuse and pain-catastrophizing, whereas, contrarily, left-sided pain was associated with anxiety and kinesiophobia. Gender did not influence this pattern of association, although gender effects on lateralization have been described [8]. The present results indicate a pathway from neuropathic pain to altered affectivity and somatization, dependent on laterality, and thus a rather anatomical rather than psychological basis of brain–body interaction in osteoarthritis-related knee pain. Our results contradict Min et al. [16], who found a preferred involvement of the right hemisphere in somatization, and Bourne et al. [29], who reported right lateralization of trait anxiety, except in the presence of high arousal, which proved to be associated with weaker lateralization to the right or even lateralization to the left. Likewise, clinical data show the involvement of the left hemisphere in fear responses [12]. Aroused emotional stimuli are subject to rapid and unaware processing by the right amygdala [39], whereas the left amygdala operates on a higher level of discrimination of contextual factors [12,40]. Although we are unable to depict the levels of arousal for the patients in the present study, we do highlight the point in time when data were collected and its proximity to the operation. To this end [33], the subscale anxiety of the BSI-18 reflects autonomic arousal (e.g., nervousness, feeling tense, spells of terror or panic and restlessness). Hence, this finding is somewhat contradictory to the results of Bourne et al. [29]; however, those authors specify their findings of left hemispherical processing of high levels of arousal in response to fear as restricted to social anxiety. The prospect of knee or any other major surgery is a different stimulus known to expose respective candidates to enormous stress, likely causing arousal to some individual extent [41]. Moreover, our finding of left lateralization of somatization, and right lateralization of anxiety, is congruent with traditional psychoanalytic hypothesizing, according to which the right hemisphere fulfills the duty of processing negative affectivity [16]. However, patterns of lateralization may vary, especially under stress, and also change dependent on the temporal distance to the stimulus [6]. In addition to syndromal psychopathological aspects, the present study reports the association of childhood physical abuse with right-sided knee pain. Childhood trauma is viewed as a potentially hazardous influence on neural structures and has been reported to be associated with left-sided losses of cortical volume [42]. Apart from structural “wounds” and ensuing functional distortions related to childhood trauma, the plasticity of pain-related brain structures may result from chronic pain itself. The intensity of chronic pain related to osteoarthritis is variable, depending on the localization and the stage of the disease. Skou et al. [43] attributed these characteristics to the plasticity of the CNS, with special emphasis placed on the processes of sensitization, showing that repeated arthroplasty of the knee could result in more and wide-spread pain. Moreover, those authors report linearity of the relationship between temporal summation and the duration of knee pain. Temporal summation refers to a multitude of action potentials and the respective neuronal firing that results in an increase in neural signaling and increased hypersensitivity to pain [44]. The associated phenomena such as allodynia and hyperalgesia are coupled with psychopathology, e.g., depression [45], anxiety [46] and somatization [47]. Given the contribution of the amygdala to allodynia and hyperalgesia, on the one hand, and the lateralized pattern of psychosomatic epiphenomenology associated with it, on the other, psychopathology in relation to osteoarthritis-related knee pain is possibly reflective of the functional lateralization linked to the left and right central nucleus of the amygdala [48].

On the contrary, the valence hypothesis [4] assumes differential hemispheric control of positive and negative emotions, with the latter being processed by the right hemisphere. Accordingly, with respect to posttraumatic suffering, Spivak et al. [49] found an association of the severity of PTSD-symptoms with rightward lateralization, and Asbjornsen [14] alike reported right hemispherical involvement in PTSD. As a stress-related disorder, PTSD may reflect some of the mechanisms involved in postoperative maladaptation and, thus, could be exemplary for the individual perioperative burden of pain and the accompanying psychosomatic suffering. As to dissociation, however, the present results do not suggest its involvement in TKA-related processes of lateralization. This does not preclude the relevance of dissociation in the perioperative setting, but dissociative symptoms, such as amnesia, have been reported to be processed bihemispherically [20]. Notwithstanding, the right hemisphere may be associated with emotional reactions in the frame of somatization [16], which would not be congruent with the present study unless emotional reactions to somatization would display a pattern of lateralization different from somatization. In addition, the left hemisphere could be involved in reappraisal processes, rather than maintaining the sensation of pain [19]. Indeed, the present results are indicative of a link between the left hemisphere and pain-catastrophizing, a typical cognitive construct, on the one hand, but not with kinesiophobia, on the other, which is no less a cognitive construct. Apart from the magnitude of the significance of the relationship, which is lower for pain catastrophizing than for kinesiophobia, in this study, we suggest the co-lateralization of anxiety and kinesiophobia to reflect a covariation of anxiety and the somatic focus associated with kinesiophobia.

Kinesiophobia is the fear of movement and reinjury and involves a focus on bodily perceptions. Hence, as discussed above, this somatic focus may reflect some arousal symptoms preferably bound for right-hemispherical transmission [29]. That said, these findings are inline with the assumption of a right hemispheric focus on the maintenance of the affective state [19].

The effect of childhood trauma on the brain is believed to cause lesions, which in turn modulate the vulnerability for adult psychopathology. In that respect, Mehta et al. [3] reported a reduced volume of the left amygdala as a result of severe early institutional deprivation, which represents childhood trauma paradigmatically. In terms of lateralization, Mehta et al.’s [3] results are congruent with our finding of childhood physical abuse as a predictor of surgery on the right knee. In addition, childhood emotional abuse was reported to be associated with reduced right amygdala volume [50], explained by the authors as the result of wear and tear caused by the repetitive activation of the amygdala by stress hormones [50]. As intriguing as this neurotoxicity hypothesis is, our results link childhood physical abuse to right-sided knee pain and, therefore, suggest left hemispheric transmission. This further contradicts Mutluer et al. [20], who reported reduced sizes of the right amygdala in PTSD based on childhood abuse. Importantly, one should take into consideration that childhood trauma represents a spectrum of aversive experiences and is, therefore, not necessarily processed uniformly. In particular, physical abuse could be linked to projections different from those of emotional abuse as a consequence of the pain inevitably associated with physical violence.

To the best of our knowledge, the present study is the first to report the lateralization of osteoarthritis-related psychosomatic correlates of pain and, therefore, needs replication. This study was cross-sectional and retrospective in nature and was based on a sample of restricted size. In addition, the amount of (left/right) variance explained by the regression analysis of the present study is moderate, highlighting the potential of other factors to determine the side of affection with knee-OA. Nevertheless, the potential implications of the present findings include the possibility of an anatomical disposition to specific maladaptive syndromes in relation to OA and require further investigation in clinical and theoretical respects.

## Figures and Tables

**Table 1:** Mean (SD) of the variables of interest; OP: operation; BSI-18: brief symptom inventory-18. FDS-20: dissociative experiences scale. PCS: pain catastrophizing scale; TSK: Tampa scale of kinesiophobia; WOMAC: Western Ontario and McGill Universities arthrosis index.

	OP of the Left Knee (*N* = 78)	OP of the Right Knee (*N* = 72)	T	*p*
Childhood emotional neglect	1.88 (0.98)	1.56 (0.8)	2.26	0.03
Childhood physical abuse	1.38 (0.84)	1.5 (1.0)	−0.84	0.4
Childhood emotional abuse	1.40 (0.98)	1.40 (0.89)	0.05	<1
Childhood sexual abuse	1.17 (0.6)	1.08 (0.5)	1.05	0.3
Childhood physical neglect	2.36 (1.27)	2.38 (1.43)	−0.1	0.9
WOMAC A (preoperative knee-pain)	5.34 (1.86)	5.52 (2.22)	−0.53	0.6
WOMAC C (preoperative knee-function)	5.19 (1.97)	5.37 (2.43)	−051	0.6
FDS-20 (dissociation)	4.21 (7.62)	4.89 (6.46)	−0.59	0.6
BSI-18 total score	6.35 (6.14)	6.84 (7.21)	−0.44	0.7
BSI-18 somatisation	1.37 (1.89)	1.99 (2.65)	−1.65	0.1
BSI-18 Depression	2.71 (2.51)	3.05 (2.61)	−0.83	0.4
BSI-18 anxiety	2.29 (2.58)	1.79 (2.44)	−1.22	0.2
PCS total score	17.80 (12.49)	18.52 (13.09)	−0.35	0.7
TSK total score	21.79 (6.88)	19.88 (6.10)	1.81	0.07

**Table 2:** Binary regression analyses. Dependent variable: side of operation (0 = left, 1 = right). TSK: Tampa scale of kinesiophobia; BSI: brief symptom inventory-18; PCS: pain catastrophizing scale; FDS: Fragebogen zu dissoziativen Symptomen-20 (a short version of the dissociative experiences scale); Womac: Western Ontario and McMaster Universities Osteoarthritis Index.

	B	S.E.	Wald	df	Sig.	Exp (B)	CI Lower	CI Upper
Childhood emotional neglect	−0.50	0.28	3.17	1.00	0.08	0.60	0.35	1.05
Childhood physical abuse	0.64	0.31	4.14	1.00	0.04	1.89	1.02	3.49
Childhood emotioanl abuse	0.07	0.36	0.04	1.00	0.84	1.08	0.53	2.16
Childhood sexual abuse	−0.85	0.47	3.28	1.00	0.07	0.43	0.17	1.07
Childhood physical neglect	−0.01	0.17	0.00	1.00	0.95	0.99	0.71	1.38
Gender	−0.09	0.43	0.04	1.00	0.84	0.92	0.40	2.11
Womac pain	0.04	0.17	0.06	1.00	0.80	1.04	0.75	1.44
Womac function	−0.03	0.15	0.05	1.00	0.83	0.97	0.73	1.29
TSK total score	−0.13	0.04	9.38	1.00	0.002	0.88	0.81	0.96
BSI-18 Somatization	0.52	0.19	7.22	1.00	0.007	1.68	1.15	2.45
BSI-18 depression	0.12	0.13	0.81	1.00	0.37	1.13	0.87	1.45
BSI-18 anxiety	−0.65	0.18	12.61	1.00	0.000	0.52	0.36	0.75
PCS total score	0.05	0.03	4.06	1.00	0.04	1.05	1.00	1.10
FDS total score	0.02	0.04	0.21	1.00	0.65	1.02	0.95	1.09

## Data Availability

Not applicable.
